# Association between anesthesia assistance and precancerous lesions and early cancer detection during diagnostic esophagogastroduodenoscopy: a propensity score-matched retrospective study

**DOI:** 10.3389/fmed.2024.1389809

**Published:** 2024-07-24

**Authors:** Yang Liu, Kaier Gu

**Affiliations:** Department of Gastroenterology and Hepatology, The First Affiliated Hospital of Wenzhou Medical University, Wenzhou, Zhejiang Province, China

**Keywords:** anesthesia assistance, upper gastrointestinal cancer, precancerous lesions, esophagogastroduodenoscopy, detection rate, propensity score matching

## Abstract

**Background:**

Esophagogastroduodenoscopy (EGD) is a fundamental procedure for early detection of upper gastrointestinal (UGI) cancer. However, limited research has been conducted on the impact of sedation during EGD on the identification of precancerous lesions and early cancer (EC). This retrospective study aims to evaluate whether sedation during EGD can improve the detection rates of precancerous lesions and EC.

**Methods:**

In this propensity score-matched retrospective study, we examined medical records from outpatients who underwent diagnostic EGD at a large tertiary center between January 2023 and December 2023. Data on endoscopic findings and histology biopsies were obtained from an endoscopy quality-control system. The primary objective was to compare the rates of detecting precancerous lesions and EC in patients who received sedation during EGD vs. those who did not receive sedation. Additionally, we aimed to identify factors influencing these detection rates using binary logistic regression analysis.

**Results:**

Following propensity score matching, a total of 17,862 patients who underwent diagnostic EGD with or without propofol sedation were identified. The group that received sedation exhibited a higher detection rate of precancerous lesions and EC in comparison to the non-sedated group (1.04 vs. 0.75%; *p* = 0.039). Additionally, within the sedated group, there was an increased likelihood of identifying precancerous lesions and EC specifically at the gastric antrum (0.60 vs. 0.32%, *p* = 0.006). Binary logistic regression analysis demonstrated that independent risk factors influencing the detection rates included age, gender, observation time, and number of biopsies conducted during the procedure.

**Conclusion:**

Anesthesia assistance during EGD screening proved advantageous in detecting EC as well as precancerous lesions. It is crucial for endoscopists to consider these factors when performing EGD screening procedures.

## 1 Introduction

Upper gastrointestinal (UGI) cancers, mainly esophageal and gastric cancers, are common causes of cancer death worldwide, particularly in Southeast Asia including China (1, 2). The prognosis for advanced-stage UGI cancers remains poor (3, 4); however, early detection and treatment significantly enhance the 5-year survival rates for both esophageal and gastric cancer (5–7). Therefore, there is widespread recognition that enhancing the early diagnostic efficacy of UGI cancer plays a pivotal role in improving patient outcomes (8).

It has been documented that a significant proportion of lesions initially diagnosed as low-grade intraepithelial neoplasia (LGIN) are subsequently identified as more advanced conditions, such as high-grade intraepithelial neoplasia (HGIN) or early cancer (EC), after undergoing surgical resection (9). Additionally, individuals with LGIN have been reported to have a substantially higher risk of developing gastric cancer (GC) compared to the general population, with an approximately 25-fold increase, and even eight times higher than those with undifferentiated lesions (10, 11). Therefore, the identification of lesions like LGIN plays a critical role in impeding disease progression and facilitating the timely detection of GC.

The detection of neoplasms within the upper gastrointestinal tract heavily relies on high-quality esophagogastroduodenoscopy (EGD) (12). Various elements influence the effectiveness of EGD screening including sedation administration, duration of observation, and supplementary methods like staining, image enhancement, and magnification (13). Sedated EGD has gained significant popularity for managing patient discomfort and anxiety during the procedure (14, 15). Nevertheless, unsedated EGD remains prevalent in several Asian nations due to its procedural efficiency advantages along with apprehensions regarding associated expenses and potential risks linked with sedation (16). There is a general agreement among experts in Asia regarding the utilization of sedatives during diagnostic EGD for neoplasia, as it has been proposed to enhance the identification rates of upper GI neoplasms (8). However, it should be acknowledged that there is limited substantial evidence supporting this assertion. While previous studies have shown that sedation can improve the endoscopic detection rate of precancerous lesions and EC in the upper digestive tract (17), there is a scarcity of research on the impact of anesthesia assistance (AA) on the detection rate of these conditions in patients undergoing EGD screening. Therefore, we conducted a retrospective analysis to investigate whether AA influences the detection rate of precancerous lesions and EC in patients undergoing EGD screening and identify possible pathways.

## 2 Methods

### 2.1 Study population

We obtained all electronic medical records of patients who underwent EGD screening from the Digestive Endoscopy unit at the First Affiliated Hospital of Wenzhou Medical University (Wenzhou, China). This retrospective study included patients who underwent EGD screening between January 2023 and December 2023. The primary objective of EGD screening at our institution is to detect early upper gastrointestinal cancer. Patients made their own decision regarding whether or not to undergo gastroscopic examination with anesthesia assistance after receiving comprehensive information about the benefits, risks, and limitations associated with anesthesia assistance as well as unsedated gastroscopy. No additional procedures were performed during the gastroscopic examination. Exclusion criteria included the following: inpatients, patients under the age of 18, patients with a history of upper gastrointestinal neoplasm or precancerous lesions, patients who had undergone upper gastrointestinal surgery, patients with upper gastrointestinal bleeding, patients with gastric retention, patients with multiple times of gastrointestinal endoscopy (for whom only the first time data were extracted), and patients without sufficient data (clinical diagnosis, result diagnosis, and endoscopist).

### 2.2 Variables

The data for analysis were obtained from a database of endoscopic reports, including information such as age, gender, medical history, and reasons for undergoing EGDs, whether anesthesia assistance was provided, duration of observation, number of biopsies taken during the procedure, and details in the endoscopic reports and pathological diagnosis of patients. Anesthesia assistance was administered by anesthesiologists following standard guidelines using propofol. The patients were divided into two groups based on whether they received anesthesia assistance or not. The EGD screenings were conducted by 69 experienced endoscopists who had performed at least 1,000 examinations and had a minimum of 1 year experience prior to this study. Endoscopist experience was determined by the number of years since independently performing EGDs. The observation time referred to the duration between capturing the initial and final images. The biopsy count represented the total number of biopsies taken during each endoscopic procedure. In this study, we focused on UGI neoplasia located specifically in the esophagus and stomach while temporarily excluding the duodenum. All identified lesions were categorized into distinct groups based on their anatomical subsites as documented in the endoscopic reports. These included the esophagus, cardia, gastric fundus, gastric body, gastric angulus, gastric antrum, and gastric pylorus. The pathological diagnosis encompassed precancerous lesions and EC exclusively. Precancerous lesions referred to pathological changes closely associated with UGI neoplasia and comprised of intraepithelial neoplasia (IN). IN was further classified into LGIN equivalent to mild and moderate dysplasia, as well as HGIN equivalent to severe dysplasia. In cases where mucosal lesions suspected to be neoplastic were identified during diagnostic EGD screening, biopsies were performed. However, for biopsy results indicating HGIN without clear distinction between mucosal or submucosal involvement under diagnostic EGD screening, subsequent endoscopic submucosal dissection (ESD) with confirmatory pathological results was required. For esophageal cases specifically, a diagnosis of EC was established only if the ESD pathology confirmed cancer confined solely within the mucosal layer. Similarly, for stomach-related cases, a diagnosis of EC was made when ESD pathology indicated cancer limited to either the mucosa or submucosa layer. All pathological findings originated from resected specimens or biopsies and underwent analysis and diagnosis by two experienced pathologists.

### 2.3 Primary and secondary outcomes

The main objective of this study was to compare the rates of detecting precancerous lesions and EC between a group that received non-anesthesia assistance and another group that received anesthesia assistance, after conducting propensity score matching (PSM) analysis. Additionally, we aimed to determine the factors that influence the detection rate of precancerous lesions and EC through binary logistic regression analysis.

### 2.4 Statistical analysis

The sample size was determined based on the available data of patients who underwent EGD screening at a medical facility in Wenzhou, China from January 2023 to December 2023. The decision to not statistically calculate the sample size was due to the inability to determine the necessary parameters in advance without references. However, it should be noted that an adequate number of cases were collected for conducting an exploratory study. To assess normality, continuous variables were verified using the Kolmogorov–Smirnov test. Subsequently, appropriate analysis techniques such as *t*-test or ANOVA were used for normally distributed variables, while skewed distributions were analyzed using Mann–Whitney U-test or Kruskal-Wallis test. Results are reported as mean ± standard deviation (SD) or median with interquartile range (IQR), respectively. Categorical variables underwent scrutiny through Fisher’s exact test or chi-square tests and are presented as number (percentage). PSM was employed to compare the baseline characteristics of two groups and minimize potential confounding variables. The propensity score, representing the likelihood of receiving anesthesia assistance, was determined through logistic regression analysis considering age, gender, and endoscopist seniority as matching variables since they influence the probability of receiving anesthesia assistance. Patients were matched at a 1:1 ratio using nearest neighbor method with a caliper set at 0.00002 of logit value derived from propensity score calculation. Paired chi-square and paired rank-sum tests were conducted in the propensity-matched cohort to compare paired groups. A binary logistic regression model was utilized to identify risk factors affecting detection rate of Precancerous lesions and EC; all variables were adjusted using enter method in this analysis to assess any association.

## 3 Results

### 3.1 Study population

**Figure 1:**
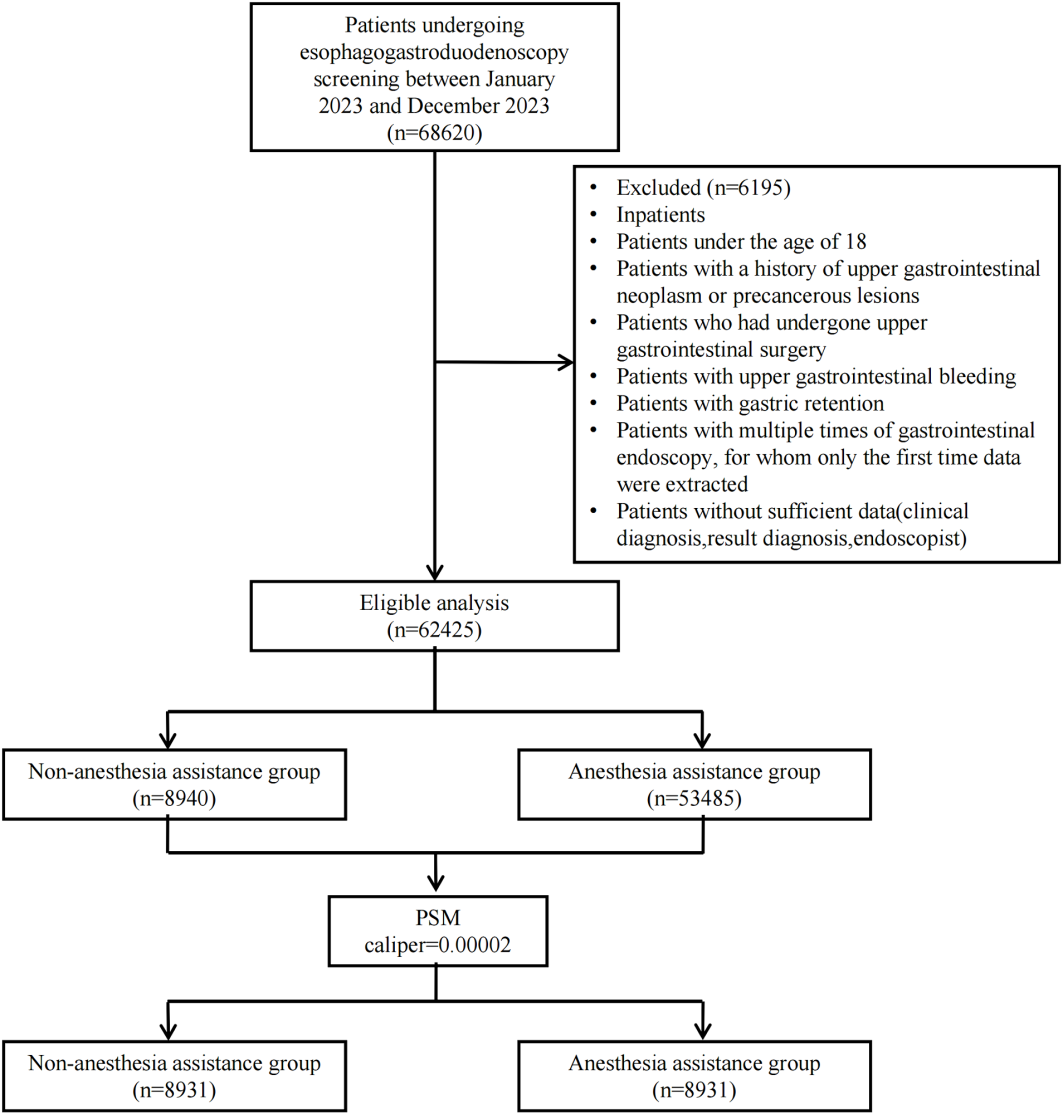
Flow diagram of the study population. PSM, Propensity score matching.

Between January 2023 and December 2023, a consecutive series of 68,620 outpatients underwent EGD at the Digestive Endoscopy unit of Wenzhou Medical University’s First Affiliated Hospital in China. A total of 62,425 patients were ultimately included in this study: 8,940 received non-anesthesia assistance while 53,485 received anesthesia assistance. Following PSM with a caliper value of 0.00002, the final analysis comprised a cohort of 17,862 patients (8,931 in each group). Figure 1 illustrates the patient distribution.

### 3.2 Baseline data and PSM

Table 1 presented the baseline characteristics of patients prior to and following propensity score matching. The findings indicated notable disparities in age and gender distribution between the two groups. The anesthesia assistance group exhibited a higher proportion of younger individuals and females, while males outnumbered females in both groups. Due to the lack of information on hypertension, diabetes mellitus, smoking, alcohol consumption, proton pump inhibitor (PPI) medication usage, and *H. pylori* infection status in outpatient records, these factors were not considered in the comparative analysis of sedation outcomes during EGD. Our evaluation of the collected data took into account age, gender, and endoscopist seniority as adjustment variables. Following PSM, each group consisted of 8,931 patients for subsequent analysis.

**Table 1:** Baseline characteristics of patients before and after propensity score matching.

	All patients			Propensity score-matched patients		
Variable	Non-anesthesia assistance group (*n* = 8,940)	Anesthesia assistance group (*n* = 53,485)	*p* value	Non-anesthesia assistance group (*n* = 8,931)	Anesthesia assistance group (*n* = 8,931)	*p* value
Patient factors						
Age, year [median (IQR)]	53 (18)	51 (18)	<0.001	53 (18)	53 (18)	1.000
Gender, male (%)	4,584 (51.28)	26,787 (50.08)	0.037	4,577 (51.25)	4,577 (51.25)	1.000
Endoscopist seniority			<0.001			1.000
Experience < 5	2,508 (28.05)	13,031 (24.36)		2,504 (28.04)	2,504 (28.04)	
5 ≤ Experience<10	4,043 (45.22)	20,691 (38.69)		4,039 (45.22)	4,039 (45.22)	
10 ≤ experience	2,389 (26.72)	19,763 (36.95)		2,388 (26.74)	2,388 (26.74)	

### 3.3 Anesthesia assistance and detection rates of precancerous lesions and EC

Differences in the endoscopic observations of the diagnostic outcomes between the groups with and without anesthesia assistance are presented in Table 2. Out of a total of 17,862 patients, there were eight cases (0.04%) of esophageal low-grade intraepithelial neoplasia (ELGIN), 11 cases (0.06%) of esophageal high-grade intraepithelial neoplasia (EHEC), four cases (0.02%) of esophageal early cancer (EEC), 95 cases (0.53%) of gastric low-grade intraepithelial neoplasia (GLGIN), 34 cases (0.19%) of gastric high-grade intraepithelial neoplasia (GHGIN), eight cases (0.04%) of gastric early cancer (GEC), 103 cases (0.58%) of LGIN, 45 cases (0.25%) HGIN, 12 cases (0.07%)of EC, 148 cases (0.83%)of precancerous lesions, and160 cases (0.90%)of precancerous lesions and EC.

**Table 2:** Diagnostic outcomes of PSM groups.

Lesion types	Non-anesthesia assistance group (*n* = 8,931)	Anesthesia assistance group (*n* = 8,931)	OR (95% CI)	*p* value
ELGIN	4 (0.04)	4 (0.04)	1.000 (0.250–4.000)	1.000
EHGIN	4 (0.04)	7 (0.08)	1.751 (0.512–5.982)	0.366
EEC	4 (0.04)	0 (0)	0.500 (0.493–0.507)	0.134
GLGIN	39 (0.44)	56 (0.63)	1.439 (0.955–2.167)	0.080
GHGIN	14 (0.16)	20 (0.22)	1.430 (0.722–2.832)	0.303
GEC	2 (0.02)	6 (0.07)	3.001 (0.606–14.874)	0.289
LGIN	43 (0.48)	60 (0.67)	1.398 (0.944–2.070)	0.093
HGIN	18 (0.20)	27 (0.30)	1.502 (0.826–2.728)	0.179
EC	6 (0.07)	6 (0.07)	1.000 (0.322–3.102)	1.000
Precancerous lesions	61 (0.68)	87 (0.97)	1.430 (1.030–1.987)	0.032
Precancerous lesions and EC	67 (0.75)	93 (1.04)	1.392 (1.016–1.908)	0.039

ELGIN, Esophageal low-grade intraepithelial neoplasia; EHGIN, Esophageal high-grade intraepithelial neoplasia; EEC, Esophageal early cancer; GLGIN, Gastric low-grade intraepithelial neoplasia; GHGIN, Gastric high-grade intraepithelial neoplasia; GEC, Gastric early cancer; LGIN, Low-grade intraepithelial neoplasia; HGIN, High-grade intraepithelial neoplasia; EC, Early cancer; PSM, Propensity score matching; OR, Odds ratio; CI, Confidence interval.

In the propensity-matched cohort, there were no statistically significant differences observed in the detection rates of LGD (0.48 vs. 0.67%), HGIN (0.20 vs. 0.30%), and EC (0.07 vs. 0.07%) between the non-anesthesia assistance group and anesthesia assistance group. However, when considering all types of precancerous lesions combined, it was found that the anesthesia assistance group exhibited a significantly higher detection rate compared to the non-anesthesia assistance group (0.97 vs. 0.68%, *p* = 0 0.032). Similarly, when including both precancerous lesions and EC together, the anesthesia assistance group also demonstrated a significantly higher detection rate than the non-anesthesia assistance group (1.04 vs. 0.75%, *p* = 0.039).

Assisting with anesthesia during EGD was found to have a significant correlation with the presence of precancerous lesions (OR 1.430, 95% CI 1.030–1.987, *p* = 0.032) and both precancerous lesions and EC (OR 1.392, 95% CI 1.016–1.908, *p* = 0.039) at the EGJ site in this study. Similarly, other findings at the EGJ, such as EHGIN (OR, 1.751; 95% CI, 0.512–5.982; *p* = 0.366), GLGIN (OR, 1.439; 95% CI, 0.955–2.167; *p* = 0.080), GHGIN (OR, 1.430; 95% CI, 0.722–2.832; *p* = 0.303), and GEC (OR, 3.001; 95% CI, 0.606–14.874; *p* = 0.289), were also more frequently detected in the anesthesia assistance group, although the differences therein between the two groups was not significant. Findings in the esophagus, such as ELGIN (OR, 1.000; 95% CI, 0.250–4.000; *p* = 1.000), and EEC (OR, 0.500; 95% CI, 0.493–0.507; *p* = 0.134), did not differ between the two groups.

### 3.4 Anesthesia assistance and observation time and the number of biopsies

In the matched cohorts, the anesthesia assistance group had a longer observation time [171 (105) s] compared to the non-anesthesia assistance group [165 (88) s, *p* < 0.001, Table 3]. Additionally, there was a significant difference in the number of biopsies per patient between the anesthesia assistance group [1 (1)] and the non-anesthesia assistance group [1 (0), *p* = 0.006].

**Table 3:** Observation time and biopsy number in PSM groups.

	Non-anesthesia assistance group (*n* = 8,931)	Anesthesia assistance group (*n* = 8,931)	*p* value
Observation time, second [median (IQR)]	165 (88)	171 (105)	<0.001
Biopsy number per patient, *n* [median (IQR)]	1 (0)	1 (1)	0.006

PSM, Propensity score matching; IQR, Interquartile range.

### 3.5 Anesthesia assistance and precancerous lesions and EC in different anatomic subsites

Among all the precancerous lesions and EC, 23 (0.13%) lesions were detected in the esophagus, 3(0.02%) in the cardia, 0 (0%) in the gastric fundus, 19 (0.11%) in the gastric body, 33 (0.18%) in the gastric angular, 83 (0.46%) in the gastric antrum, and 0 (0%) in the gastric pylorus (Table 4). In both the groups of anesthesia assistance and non-anesthesia assistance, the majority of precancerous lesions and EC were detected in the gastric antrum. Utilizing anesthesia assistance during the procedure was found to be associated with an enhanced ability to detect precancerous lesions and EC at the gastric antrum (0.60 vs. 0.32%; odds ratio, 1.867; 95% confidence interval, 1.188–2.935; *p* = 0.006). There were no notable disparities observed in the detection rates of precancerous lesions and EC at other sites between the groups receiving anesthesia assistance and those without it.

**Table 4:** Anatomic subsites of precancerous lesions and EC detected by esophagogastroduodenoscopy in PSM groups.

Anatomic subsite	Non-anesthesia assistance group (*n* = 8,931)	Anesthesia assistance group (*n* = 8,931)	OR (95% CI)	*p* value
Esophagus	12 (0.13)	11 (0.12)	0.917 (0.404–2.078)	0.835
Cardia	1 (0.01)	2 (0.02)	2.000 (0.181–22.063)	1.000
Gastric fundus	0 (0)	0 (0)		
Gastric body	8 (0.09)	11 (0.12)	1.375 (0.553–3.421)	0.491
Gastric angular	17 (0.19)	16 (0.18)	0.941 (0.475–1.864)	0.862
Gastric antrum	29 (0.32)	54 (0.60)	1.867 (1.188–2.935)	0.006
Gastric pylorus	0 (0)	0 (0)		

EC, Early cancer; PSM, Propensity score matching; OR, Odds ratio; and CI, Confidence interval.

### 3.6 Logistic regression analysis for detection rate of precancerous lesions and EC

In the propensity-matched cohort, we conducted univariate analyses to identify variables that influenced the detection rate of precancerous lesions and EC. The results indicated that age, gender, observation time, and the number of biopsies were all statistically significant factors (*p* < 0.05). Subsequently, a binary logistic regression model was developed to assess the association between anesthesia assistance and the detection rate of precancerous lesions and EC (Table 5). However, after adjusting for other factors, there was no significant correlation found between anesthesia assistance and the detection rate of precancerous lesions and EC (95% CI, 0.970–1.840; *p* = 0.076). Interestingly, the detection rate of precancerous lesions and EC in elderly patients is significantly higher than in younger patients. For each additional year of a patient’s age, the likelihood of detecting precancerous lesions and EC increases is 1.064 times the original (95% CI: 1.051–1.082; *p* < 0.001). Moreover, male patients had a significantly higher detection rate than female patients with a relative risk of 1.749 (95% CI: 1.275–2.520; *p* = 0.001). Additionally, for every additional second of observation time during endoscopy procedures, the likelihood of detecting precancerous lesions and EC increases is 1.001 times the original (95% CI: 1.001–1 0.002; *p* < 0 0.001). Similarly, with each additional biopsy, the detection rate of detecting precancerous lesions and EC increased by 2.003-fold (95 %CI: 1.615–2.485; *p* < 0.001).

**Table 5:** Logistic regression analysis for detection rate of precancerous lesions and EC in PSM groups.

Anatomic subsite	Unadjusted OR	95% CI	*p* value	Adjusted OR	95% CI	*p* value
Anesthesia assistance						
Without anesthesia assistance	Reference			Reference		
With anesthesia assistance	1.392	1.016–1.908	0.040	1.336	0.970–1.840	0.076
Patient factors						
Age	1.080	1.065–1.095	<0.001	1.067	1.051–1.082	<0.001
Gender	2.106	1.506–2.946	<0.001	1.792	1.275–2.520	0.001
Endoscopist seniority						
Experience<5	Reference		0.347	Reference		0.554
5 ≤ experience<10	1.259	0.848–1.870	0.253	1.250	0.836–1.868	0.277
10 ≤ experience	1.364	0.887–2.098	0.158	1.155	0.745–1.788	0.520
Observation time	1.002	1.001–1.002	<0.001	1.001	1.001–1.002	<0.001
Biopsy number	3.082	2.530–3.754	<0.001	2.003	1.615–2.485	<0.001

EC, Early cancer; PSM, Propensity score matching; OR, Odds ratio; and CI, Confidence interval.

## 4 Discussion

In this retrospective analysis, after employing propensity score matching, we discovered no significant difference in the identification rate of precancerous lesions and EC between patients who received assistance from anesthesia and those who did not. After adjusting for other factors, age, gender, duration of observation time, and the number of biopsies were all independent risk factors affecting the detection rate of precancerous lesions and EC.

Esophagogastroduodenoscopy is widely recognized as a highly effective method for diagnosing neoplasms in the esophagus, stomach, and duodenum. However, it is important to note that not all cancers in the UGI tract are initially detected during this procedure. Several studies have indicated that missing gastric cancer with EGD is not uncommon, with a potential miss rate of 5–10% (18, 19). Additionally, research has shown that endoscopically missed gastric cancer (lesions diagnosed within 12 months after the initial EGD) and metachronous gastric cancer (lesions detected more than 1 year after the initial EGD) occur in less than 26% (20) and 16% (21) of surveillance EGDs, respectively. A prospective study conducted on an Australian population revealed a missed cancer rate of 4.8% (95% CI 2.1–10.4) during EGD screenings (22). A meta-analysis of international studies found that the prevalence of UGI cancer detection during EGD within the past 3 years was low, with estimates ranging from 7.5 to 16.6%. However, there were variations in these estimates across different studies (23). On average, one case of UGI cancer may be missed for every 400 gastroscopies performed. Therefore, it is crucial to improve the quality of EGD screening procedures to overcome these limitations effectively. The potential for undetected cancers has been correlated with factors that may be connected to the technical execution of EGD. Undetected cancers have been linked to smaller tumor sizes (24), flat or depressed appearances (19), positioning in the gastric body or posterior wall (18, 19, 25, 26), limited experience of endoscopists (20, 27), shorter periods of observation (28), and an insufficient number of biopsies (18).

Esophagogastroduodenoscopy performed solely with topical pharyngeal anesthesia often leads to adverse reactions like nausea, vomiting, and general discomfort in the majority of patients. Numerous approaches have been employed to enhance EGD tolerance, including the use of intravenous sedation and smaller endoscopes. The main goals of sedation administration during EGD are to reduce patient anxiety and discomfort, promote patient cooperation, and improve satisfaction for both patients and endoscopists, ultimately enhancing the quality of the procedure (29). Based on our clinical experience, it has been observed that the utilization of sedation during EGD procedures may increase the likelihood of detecting precancerous lesions and EC. A study conducted at multiple centers found that the utilization of propofol as an anesthesia adjunct resulted in improved identification rates of superficial neoplasms among patients who underwent EGD screening (30). Similarly, another multicenter study demonstrated that the presence of anesthesia support increased the detection rate of early cancer and precancerous lesions during EGD screening (17). Wu et al. also observed a significantly elevated detection rate of small upper gastrointestinal neoplasms when anesthesia assistance was provided during EGD screening (31). Furthermore, a retrospective observational study reported that performing sedated EGD led to an improvement in identifying small lesions, particularly polyps measuring 5 mm (32). Zhou et al. discovered that the presence of anesthesia assistance could potentially enhance the identification of early cancer and HGIN during endoscopic procedures. They further observed that the presence of anesthesia support could potentially enhance the identification rate of EC and HGIN in the upper gastrointestinal tract, potentially through enabling the utilization of additional endoscopic methodologies, prolonging examination duration, and acquiring biopsies from diverse sites (17). However, Lee et al.’s study revealed no significant impact of anesthesia assistance on the detection rate of early gastric cancer (33). In this study, our findings indicate that the utilization of anesthesia assistance during EGD procedures can potentially improve the identification rate of precancerous lesions and EC. However, after considering other factors, binary logistic regression analysis did not reveal a significant association between anesthesia assistance and the detection rate of precancerous lesions and EC. Age, gender, observation time, and the number of biopsies were identified as independent factors influencing the detection rate of precancerous lesions and EC in this study. There are various potential reasons for the enhancement of identifying precancerous lesions and EC through sedation. Firstly, sedation can enhance patient comfort and their ability to endure the EGD procedure (29). Improved patient cooperation greatly benefits endoscopic examinations as it allows for adequate air insufflation, a steady field of vision, and extended inspection time—all crucial factors contributing to the quality of EGD (12).

A study has demonstrated that specialized training can increase the endoscopic detection rate of early gastric cancer (34). Additionally, a multicenter retrospective study has indicated that undergoing a systematic training course can lead to an improvement in detecting early gastric cancer cases (35). However, our findings contradict this notion as we discovered that the experience level of endoscopists does not impact the detection rate of precancerous lesions and EC.

Endoscopists who perform rapid examinations may fail to detect neoplastic lesions in the upper gastrointestinal tract due to insufficient observation time (36). Studies have shown that endoscopists who take their time during examinations are more likely to detect neoplasms compared to those who work quickly (37). Prolonged examination periods have been found to increase the detection rate of neoplasms in EGD screening (38). Other research suggests that setting a minimum examination time of 6 min can enhance the identification of focal UGI tract lesions during EDG procedures (39). Teh et al. discovered that extending the observation time improves the rate at which lesions are detected, particularly for endoscopists who spend an average of 7 min or more on each diagnostic EGD examination (40). Our study revealed that for every additional second of observation time during endoscopy procedures, the likelihood of detecting precancerous lesions and EC increases is 1.001 times the original. It has been reported that sedation during EGD is associated with longer observation times, potentially contributing to improved lesion detection rates through extended examinations (17). Therefore, sedation is considered beneficial for thorough inspection of the UGI tract.

Furthermore, the likelihood of obtaining an accurate pathological diagnosis is enhanced by acquiring sufficient tissue samples from concerning lesions during an EGD (12). A multicenter cohort study also discovered that endoscopists who performed biopsies more frequently had fewer instances of undetected cancer and identified a higher number of precancerous conditions in the stomach (41). Our findings revealed an increased biopsy rate in the group receiving anesthesia assistance. We believe that improved examination with anesthesia assistance may result in more precise biopsies of suspected lesions, leading to a higher detection rate of these lesions.

The present study evaluated and quantified the location of precancerous lesions and EC, indicating that the anesthesia assistance group exhibited higher detection rates for gastric antrum precancerous lesions and EC compared to the non-anesthesia assistance group, although there were no significant differences between the two groups in this regard. Our findings indicate that the presence of anesthesia assistance significantly enhances the ability to detect GLGIN, while there is no significant impact on the detection rates of ELGIN. The stomach, particularly its great curvature, contains numerous folds that necessitate air insufflation for thorough examination. However, in unsedated patients, the occurrence of flatulence and belching resulting from air insufflation can disrupt focused observation. Consequently, sedation plays a crucial role in ensuring successful and comprehensive endoscopy.

This study employed an observational and retrospective approach, encompassing a substantial sample size. Several measures were implemented to mitigate bias. We specifically excluded therapeutic EGDs, focusing solely on diagnostic EGDs to assess the diagnostic yield of sedated and unsedated approaches. Furthermore, we only included highly skilled endoscopists with extensive experience (over 1 year) and a significant number of prior EGDs performed (more than 1,000), aiming to minimize any potential influence they may have had on the examination outcomes. To effectively control for confounding variables, we utilized PSM, which is considered one of the most rigorous methods for approximating randomized trials within retrospective designs. Following PSM implementation, the resulting cohorts demonstrated excellent balance, thereby mitigating any bias stemming from differences in morbidity between them. Lastly, we conducted a comprehensive analysis considering various factors in accordance with guidelines and our clinical expertise to explore potential mechanisms.

Despite the strengths mentioned, it is important to acknowledge several limitations in the current study. Firstly, due to its retrospective design, there may be challenges in collecting certain influencing factors such as patients’ dietary habits, exercise routines, smoking status, obesity levels, presence of chronic airway diseases, usage of PPI medication, *H. pylori* infection status, and family history of upper GI neoplasms. Although all endoscopic information was carefully recorded prospectively, potential bias cannot be completely ruled out. Therefore, future studies should consider conducting prospective randomized case–control trials to address these limitations. Secondly, while we categorized patients into two groups based on whether they received anesthesia assistance or not during the procedure; unfortunately, we did not collect data on the reasons behind their choice. This lack of information could introduce selection bias and potentially impact the external validity of our findings. Thirdly, this study did not document any adverse events related to sedation during the procedures, which resulted in a lack of essential safety data for this aspect of patient care. Evaluating sedation critically involves considering its complications as well. It is worth noting that no major complications such as aspiration pneumonia or respiratory distress were observed among all patients who underwent sedative endoscopy procedures. Fourthly, this study lacks follow-up information, which prevents us from comparing missed cancer rates between sedated and unsedated EGDs. Fifthly, it is important to note that this study was conducted at a single medical center, which may introduce inherent selection bias. Therefore, caution should be exercised when generalizing the findings of this study to other medical centers. Further validation is required to confirm their applicability. Finally, it is worth mentioning that only the Chinese population was included in this study. Considering the varying prevalence of UGI tract cancers across different countries, it is important to acknowledge that these findings may not accurately reflect the situation worldwide. The potential advantages of sedation in regions with a lower incidence of cancer in the upper gastrointestinal tract might be relatively diminished. Therefore, further investigations are necessary for nations where UGI tract cancers are less prevalent.

## 5 Conclusion

In summary, the detection rate of precancerous lesions and EC in patients undergoing EGD screening does not reveal a significantly different when anesthesia assistance is provided compared to when it is not. However, sedation during the procedure may enhance the identification of precancerous lesions and EC in the upper digestive tract by allowing for longer observation time and increased biopsies from various locations. It is important for endoscopists to consider these factors when performing EGD screening. Our findings support the promotion and focus on sedative endoscopy and its impact on quality control during endoscopic procedures. Considering its cost-effectiveness and convenience in East China, anesthesia assistance emerges as a preferable diagnostic option over non-anesthesia assistance. Therefore, we recommend considering sedation to improve the overall quality of EGD examinations.

## Data availability statement

The data analyzed in this study were subject to the following licenses/restrictions: the datasets used and analyzed during the current study are available from the corresponding author on reasonable request. Requests to access these datasets should be directed to KG, 805109022@qq.com.

## Ethics statement

The studies involving humans were approved by the Ethics Committee of the First Affiliated Hospital of the Wenzhou Medical University. The studies were conducted in accordance with the local legislation and institutional requirements. Written informed consent for participation was not required from the participants or the participants’ legal guardians/next of kin in accordance with the national legislation and institutional requirements.

## Author contributions

YL: Data curation, Formal analysis, Investigation, Methodology, Resources, Software, Writing – original draft. KG: Conceptualization, Supervision, Validation, Writing – review & editing.
